# Maximum flexion and lateral rollback revealed better patient satisfaction after total knee arthroplasty

**DOI:** 10.1186/s43019-024-00219-4

**Published:** 2024-03-27

**Authors:** M. Tamaki, T. Ishibashi, T. Yamazaki, S. Konda, K. Kono, S. Okada, T. Tomita

**Affiliations:** 1 Department of Orthopedics, Osaka University Graduate School of Medicine, 2-2 Yamadaoka Suita, Osaka, 565-0871 Japan; 2 Division of Orthopedic Biomaterial Science, Osaka University Graduate School of Medicine, Osaka, Japan; 3 Department of Information Systems, Faculty of Engineering, Saitama Institute of Technology, Fukaya, Japan; 4 Department of Health and Sport Sciences, Osaka University Graduate School of Medicine, Osaka, Japan; 5 Department of Orthopaedic Surgery, Faculty of Medicine, The University of Tokyo, Tokyo, Japan; 6 Graduate School of Health Sciences, Morinomiya University of Medical Sciences, Osaka, Japan

**Keywords:** Fluoroscopic analysis, Patient-reported outcomes, Femoral rollback, Total knee arthroplasty

## Abstract

**Introduction:**

Patient satisfaction is an important outcome of total knee arthroplasty (TKA). However, we cannot predict how and why patients are satisfied or dissatisfied with TKA. The hypothesis of this study was that patient-reported outcomes (PROs) correlate with in vivo kinematics after TKA.

**Materials and methods:**

One hundred knees were analyzed after TKA. The in vivo kinematics of deep knee bending motion were estimated from single-plane fluoroscopy using a two-to-three-dimensional registration technique. Active knee flexion, femoral rotation and rollback were evaluated. The PROs were obtained after surgery using the 2011 Knee Society Scoring System (KSS), and their relationship with in vivo kinematics was determined.

**Results:**

The average minimum and maximum flexion were −2.4 ± 7.3° and 113.2 ± 13.6°, respectively. The average femoral rotation was 7.4 ± 3.4°, and the average medial and lateral rollback were 2.4 ± 4.8 mm and 7.2 ± 5.6 mm, respectively. The multiple regression analysis revealed that the maximum flexion angle significantly contributed to symptoms and satisfaction. In addition, lateral rollback was also a significant factor affecting patient satisfaction. Lateral rollback and lateral Anterior-Posterior (AP) position at maximum flexion were correlated with the maximum flexion angle, whereas femoral rotation did not correlate with flexion angles.

**Conclusions:**

Maximum flexion and lateral rollback are important for better patient satisfaction after TKA. To obtain the maximum flexion angle, it was necessary to perform the normal kinematic pattern with a large amount of lateral rollback.

## Introduction

Total knee arthroplasty (TKA) is a well-accepted procedure for the treatment of end-stage knee arthritis [1]. TKA has been successful in pain relief and improving the functional status of patients. Despite advances in surgical techniques and implant design for TKA, up to 20% of patients remain dissatisfied with the outcome[2, 3]. Patient satisfaction is an important outcome because the discrepancy between the surgeon and patient ratings of mental and physical health status is well documented [2–5]. Unfortunately, predicting patient satisfaction or dissatisfaction with TKA remains challenging.

In 2011, the new Knee Society Knee Scoring System (2011 KSS) was developed to better characterize the expectations, satisfaction, and physical activities of patients who underwent TKA [6]. This patient-reported outcome can be used to balance the discrepancy between the surgeon’s objective and the patient’s subjective evaluations.

Fluoroscopic analysis of TKA was developed in the 1990s [7–10]. Currently, it is possible to accurately evaluate the flexion angle, axial rotation angle, and AP position between the femoral and tibial components during active motion [11–15]. Fluoroscopic analysis has the advantage of being able to quantitatively assess the patient’s activities of daily living and is expected to be closely related to patient-reported outcomes (PROs), through which the patient also evaluates activities of daily living. However, many post-TKA kinematics studies have reported implant-specific kinematics results as well as PROs, and few reports have discussed the relationship between various implant kinematics and PROs for TKA as a whole.

The aim of this study is to evaluate the relationship between in vivo kinematics and the 2011 KSS as PROs after various TKA implants at our institution. By analyzing kinematic data, including flexion, rotation and rollback, we aim to identify kinematic patterns associated with better PRO after TKA. Our hypothesis is that PROs will correlate with in vivo kinematics using a two-to three-dimensional registration technique.

## Material and methods

In this retrospective study, a total of 100 knees from 95 patients for whom the 2011 KSS could be obtained were analyzed among 140 knees that underwent fluoroscopic analysis more than 6 months after TKA. From March 2007 to November 2017, the knees underwent total knee arthroplasty by the same surgical team. The average age was 72.7 ± 8.0 years at operation, and 18 males and 77 females were analyzed. All patients were diagnosed with osteoarthritis, rheumatoid arthritis, and spontaneous osteonecrosis. Although both varus and valgus deformity were included in our study, exclusion criteria were flexion deformity, inability to flex more than 90 degrees, severe bone loss, and previous history of total knee replacement. The appropriate institutional review board approved this study, and written informed consent was obtained from all patients. A total of 34 knees after cruciate-retaining (CR) TKA (Triathlon; Stryker, Journey 2; Smith and Nephew, and FINE; Teijin Nakashima), 35 knees after posterior stabilized (PS) TKA (Triathlon; Stryker, Legion; Smith and Nephew, and FINE; Teijin Nakashima), 16 knees after bicruciate substituting (BCS) TKA (Journey 2; Smith and Nephew), 9 knees after bicruciate retaining (BCR) TKA (Vanguard; Zimmer Biomet), and 6 knees after cruciate substituting (CS) TKA (Triathlon; Stryker) were analyzed. The selection of the implant was dependent on the type of deformity and instability. In general, a CR prosthesis was used for medial compartment OA with a mild deformity. Posterior-stabilized and bicruciate-substituting prostheses were used for severe deformities and anterior cruciate ligament/posterior cruciate ligament (ACL/PCL) deficiency. Bicruciate-retaining implants are rarely used for limited patients. Other patients with mild-to-moderate deformities were treated with cruciate-substituting (CS) implants. All TKAs were performed using the same surgical technique by the three knee surgeons. A senior surgeon participated in all of the procedures as either the chief surgeon or first assistant. We performed the TKA procedures using the medial para‐patellar with modified gap technique for CS, PS, and BCS TKA and with the measured resection technique for CR and BCR TKA. The PCL was preserved using the bone block technique in CR TKA and the ACL and PCL were preserved in BCR TKA. For the tibia, sagittal alignment was targeted to be within 3° of the postoperative posterior tibial slope (PTS) in CS, PS, and BCS TKA and to 7° of the postoperative PTS in CR and BCR TKA, as per the manufacturers’ recommendations. The joint gaps in flexion and extension were adjusted to be equalized. If the extension gap was tight, we released the medial soft tissue and performed a reduction osteotomy. The patella was resurfaced in all cases, and all components were cemented. Postoperatively, full weight bearing was allowed the following day and range of motion and strengthening exercises were started.

### In vivo kinematic analysis of active knee flexion

**Fig. 1:**
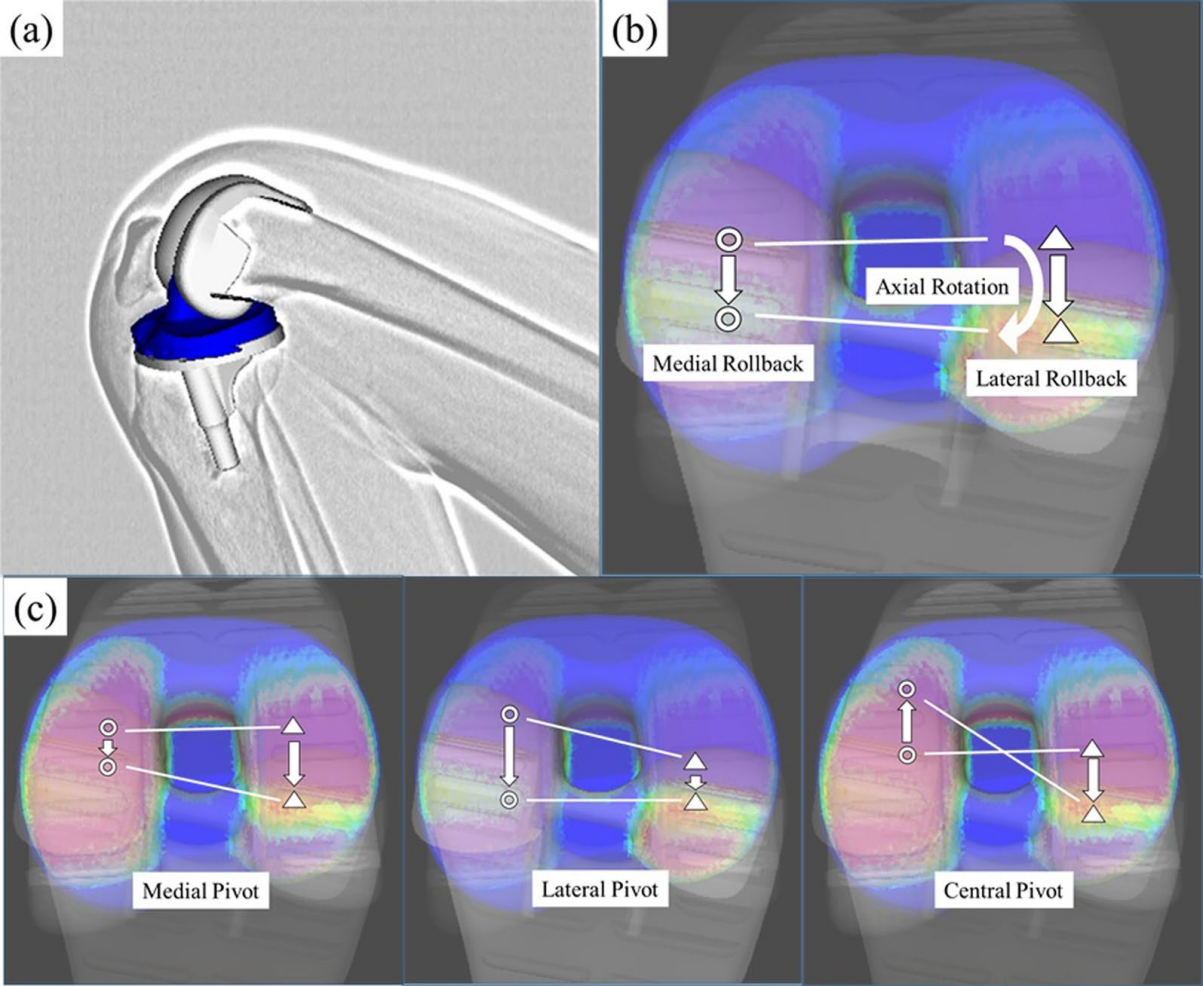
**a** The two-dimensional/three-dimensional registration technique uses computer-assisted design models to reproduce the spatial position of femoral and tibial components from single-view fluoroscopic images. **b** The axial rotation angles of the femoral component relative to the tibial component were denoted as positive from minimum flexion to maximum flexion. Medial and lateral rollbacks were defined as the AP translation of the medial and lateral sides from minimum flexion to maximum flexion. **c** A medial pivot was defined if the lateral side moved more than the medial side, the lateral pivot was defined if the medial side moved more than the lateral side, and the central pivot was defined if the medial and lateral sides moved in opposite directions

At the time of the fluoroscopic analysis, the mean duration of postoperative follow-up was 13.1 ± 11.3 months. The mean height was 155.3 ± 6.9 cm, and the mean body weight was 62.2 ± 9.7 kg. Under fluoroscopic surveillance in the sagittal plane, each patient was asked to perform sequential active knee flexion from full extension to maximum flexion, equivalent to a squatting motion, under weight-bearing conditions. (Fig. 1a) Patients were allowed to hold onto a handrail for safety purposes. Sequential active knee flexions were recorded using digital X-ray images (1024 × 1024 × 12 bits/pixel, with 7.5 Hz serial spot images as a DICOM file) with 17 inch flat-panel detector systems from two different manufacturers (C-vision Safire L; Shimadzu, Kyoto, Japan, and SIEMENS Artis zee; Siemens, Munich, Germany). These systems provided images that were undistorted and clear compared with the images provided by the intensifier systems. To estimate the spatial position and the orientation of the artificial knee prosthesis, we used a two-to-three-dimensional registration technique [9, 10]. This technique uses a contour-based registration algorithm that employs single-view fluoroscopic images and three-dimensional computer-aided design models. The estimation accuracy of the relative position between the metal components using this system was within 0.5° in rotation and 0.4 mm in translation [10]. In the femoral coordinate system, the origin is defined as the center of gravity of the component. In the tibial coordinate system, the origin is defined as the center of the tibial tray surface. The flexion and external rotation angles of the femoral component relative to the tibial component were denoted as positive from minimum flexion to maximum flexion. The AP position was defined as the point nearest to the femoral component of the tibial axial plane on both the medial and lateral sides. Positive or negative values of AP translation were expressed as anterior or posterior translation to the axis of the tibial component, respectively. Medial and lateral rollbacks were defined as the AP translation of the medial and lateral sides from minimum to maximum flexion. (Fig. 1b) A medial pivot was defined if the lateral side moved more than the medial side, a lateral pivot was defined if the medial side moved more than the lateral side, and a central pivot was defined if the medial and lateral sides moved in opposite directions. (Fig. 1c).

### Patient-reported outcomes (PROs)

The 2011 KSS was used to evaluate the patient-reported outcomes. The 2011 KSS has four categories: symptoms, patient satisfaction, patient expectations, and functional activities. The functional activity item is composed of walking and standing, standard activities, advanced activities, and discretionary activities. The patients were asked to grade their symptom expectations, satisfaction, and functional activities using the Japanese version of the 2011 KSS [16]. At the time of evaluation of the 2011 KSS, the mean duration of postoperative follow-up was 24.0 ± 17.2 months.

### Statistical analysis

A post hoc power analysis was performed using G-Power 3.1 software (Institut fur Experimentelle Psychologie, Dusseldorf, Germany) to determine the power of the study considering the current sample size. Using an *α* of 0.05 and a medium effect size (*f*^2^ = 0.15), a total sample size of 68 provided a study power of 0.8. All data are expressed as the mean ± standard deviation (SD). The data from the fluoroscopic analysis and the 2011 KSS are considered continuous data. We performed a multiple linear regression analysis with a stepwise procedure to identify correlations between the data from the fluoroscopic analysis and the 2011 KSS. To identify the correlations among the data from the fluoroscopic analysis, we performed a multiple linear regression analysis with a stepwise procedure and Tukey‒Kramer analysis. Statistical significance was set at *P* < 0.05, and all analyses were performed using JMP Pro software, version 17.0.0 (SAS Institute, Inc., Cary, NC).

## Results

The data from the fluoroscopic analysis and 2011 KSS are presented in Table 1. The average minimum and maximum flexion were −2.4 ± 7.3° and 113.2 ± 13.6°, respectively. The average femoral rotation was 7.4 ± 3.4°, and the average medial and lateral rollback were 2.4 ± 4.8 mm and 7.2 ± 5.6 mm, respectively. Multiple linear regression analyses using a stepwise procedure were performed for both fluoroscopic analysis and each score from the 2011 KSS, as shown in Table 2. The multiple regression analysis revealed that the maximum flexion angle significantly contributed to symptoms and satisfaction. In addition, lateral rollback was also a significant factor affecting patient satisfaction. The range of motion and medial AP position at maximum flexion were associated with each of the functional activities. With respect to the medial AP position, a more anterior position at maximum flexion correlated with each of the functional activities. Furthermore, the minimum flexion angle was found to be a contributing factor for discretionary activities. The medial pivot pattern was observed in 69 knees, the lateral pivot pattern in 20 knees, and the central pivot pattern in 11 knees. However, there were no significant differences in symptoms, satisfaction, expectations, or functional activities according to the pivot pattern.

**Table 1:** Demographic data from the fluoroscopic analysis and 2011 Knee Society Score

	Average	SD
Minimum flexion (°)	−2.4	7.3
Maximum flexion (°)	113.2	13.6
Range of motion (°)	115.7	16.1
Minimum rotation (°)	3.2	4.5
Maximum rotation (°)	10.6	4.4
Range of rotation (°)	7.4	3.4
Medial AP at minimum flex (mm)	−1.3	3
Medial AP at maximum flex (mm)	−3.7	4.4
Medial rollback (mm)	2.4	4.8
Lateral AP at minimum flex (mm)	−5	4.9
Lateral AP at maximum flex (mm)	−12.2	4.1
Lateral rollback (mm)	7.2	5.6
Symptoms (25)	19.7	5.4
Satisfaction (40)	27	8.9
Expectations (15)	9.6	3.1
Functional activities (100)	66.7	19.9
Walking and standing (30)	20.6	8.6
Standard activities (30)	23	5.6
Advanced activities (25)	13.4	6.3
Discretionary activities (15)	9.7	4.6

**Table 2:** A multiple linear regression analysis with stepwise procedure from fluoroscopic analysis and 2011 KSS

	Independent variable	*β*	(95% CI)	*P* value	*R*^2^ value
Symptoms	Max flexion	0.09	(0.01–0.17)	0.021	0.05
Satisfaction	Max flexion	0.25	(0.1–0.4)	0.0011	
	Lateral Rollback	0.5	(0.01–0.92)	0.0177	0.11
Expectations	NA				
Functional activities	Range of motion	0.49	(0.26–0.72)	< .0001	
	Medial AP at maximum flex	1.26	(0.44–2.1)	0.003	0.18
Walking and standing	Range of motion	0.11	(0.01–0.22)	0.03	
	Medial AP at maximum flex	0.59	(0.22–0.97)	0.002	0.1
Standard activities	Range of motion	0.12	(0.06–0.19)	0.0004	
	Medial AP at maximum flex	0.29	(0.05–0.53)	0.02	0.13
Advanced activities	Range of motion	0.18	(0.11–0.25)	< .0001	
	Medial AP at maximum flex	0.31	(0.05–0.56)	0.02	0.2
Discretionary activities	Minimum flexion	−0.15	(−0.26 to −0.03)	0.01	0.06

*β* regression coefficient. *95% CI* 95% confidence interval. *R*^2^ value, correlation coefficient

**Fig. 2:**
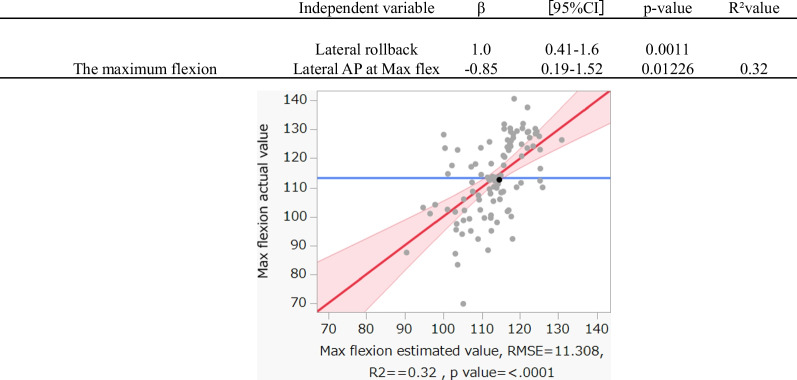
A multiple linear regression analysis with a stepwise procedure from the maximum flexion angle and other kinematic data

A multiple regression analysis using a stepwise method was performed to evaluate the relationship between the maximum flexion angle, which correlates with symptoms and satisfaction, and other kinematic data, as shown in Fig. 2. The results revealed that the lateral rollback and lateral AP position at maximum flexion were correlated with the maximum flexion angle.

**Fig. 3:**
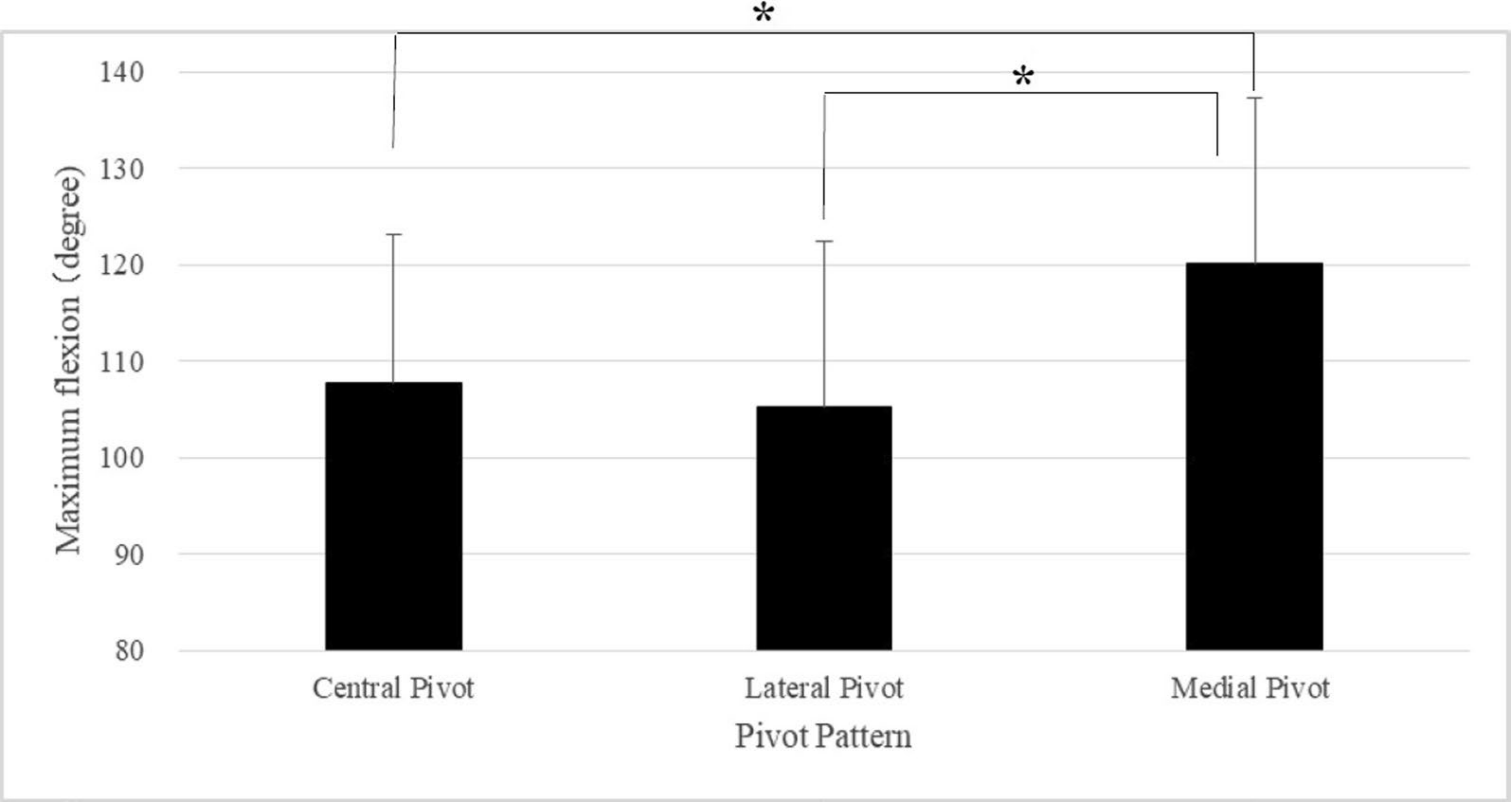
Maximum flexion for each pivot pattern. *Correlations are statistically significant (*P* < 0.05)

Considering the maximum flexion angle by the pivot pattern, the mean maximum flexion angle was 107.7 ± 15.4° for the central pivot, 105.2 ± 16.8° for the lateral pivot, and 120.1 ± 14.3° for the medial pivot. The maximum flexion angle of the medial pivot was significantly larger than that of the central and lateral pivots. (*P* < 0.05) (Fig. 3).

**Fig. 4:**
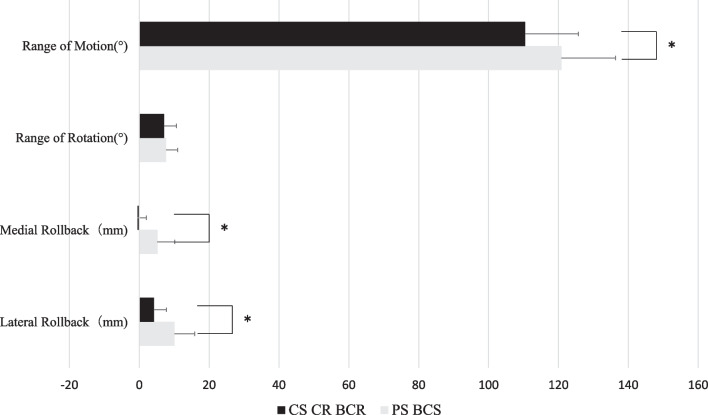
Comparison of the kinematic data between the PS and BCS and the CS, CR, and BCR with and without postcam mechanism. *Correlations are statistically significant (*P* < 0.05)

Considering the implant type, the range of motion was 120.2 ± 15.3° for PS and BCS with postcam and 110.4 ± 15.5° for CS, CR, and BCR without postcam. In addition, the medial and lateral rollback for PS and BCS were 5.2 ± 2.5 mm and 10.0 ± 3.6 mm, respectively. The medial and lateral rollback for CS, CR, and BCR were −0.5 ± 5.0 mm and 4.2 ± 5.7 mm for CS, CR, and BCR, respectively. PS and BCS implants with postcam mechanism had better range of motion and medial and lateral rollback than CS, CR, and BCR implants (Fig. 4).

## Discussion

The most significant findings of the present study were as follows: the kinematic data correlated with patient satisfaction were maximum flexion and lateral rollback, while the kinematic data that correlated with functional activities were the range of motion, including the extension angle, and the medial AP position located anterior to the maximum flexion. Furthermore, the increase in maximum flexion, which has the greatest impact on satisfaction, is achieved when the lateral rollback is larger and the lateral AP position is more posterior.

Although several studies have reported the correlation between the flexion angle and PROs, these flexion angles are often based on passive knee flexion measured in the examination room [16, 17]. In these studies, the passive knee flexion angle measured by the goniometer was inaccurate, whereas the active knee flexion measured by our fluoroscopic analysis was more accurate because the relative position between the metal components was calculated to be within 1 mm and 1°. Regarding the relationship between intraoperative kinematics using a navigation system and PROs in TKA, a previous report showed that an intraoperative medial pivot pattern led to better PROs [18]. However, to our knowledge, the relationship between in vivo kinematics and PROs has not been well studied. Recently, Kage reported that achieving medial AP translation and femoral external rotation stability in early flexion may be important for optimizing postoperative PROs [19, 24]. Another study showed that patients with poor PRO scores after TKA experience more anterior translation on the medial side followed by medial midflexion instability and less posterior translation on the lateral side in deep flexion than patients with good PROs during closed kinetic chain exercises [20]. In our study, the kinematic data correlated with patient satisfaction were maximum flexion and lateral rollback, while the kinematic data correlated with functional activities were the range of motion and the medial AP position located anterior at the maximum flexion. The results of these findings suggest that a stable anterior medial AP position and lateral rollback during maximum flexion are associated with improved PROs as patient satisfaction and functional activities Our results also show that the kinematic pattern with a large amount of lateral rollback, which moved from anterior to posterior upon flexion, was able to achieve deep knee flexion. In addition, a consideration of the pivot pattern showed that a medial pivot pattern with a larger lateral rollback than medial rollback resulted in a higher flexion. In the normal knee, external rotation and bicondylar rollback of the femur are observed with knee flexion. Previous reports of in vivo squatting motion kinematics after TKA showed approximately 10° of femoral external rotation relative to the tibia, although less than in the normal knee. It was also reported that the normal rotation pattern, in which the femur is externally rotated with knee flexion, has better weight-bearing flexion than the reverse rotation pattern, in which the femur is internally rotated [21]. Regarding femoral rollback, there are many reports of bicondylar rollback after midflexion, mainly in PS-TKA [11–14, 19]. CR TKA has also been reported to result in a paradoxical anterior translation pattern, in which the femur moves forward with midflexion [22]. The results of the present study on various implant types showed that a normal rotation pattern and lateral rollback are necessary for deep knee flexion. Previous studies have also demonstrated that patients who achieve deep flexion after TKA consistently maintain the posterior condylar contact point from extension to flexion [23]. Although this study was based on a single implant design, our study showed that deep knee flexion can be achieved with lateral rollback in flexion, even in patients who are anterior in extension with other implant designs. The lateral rollback was larger than the medial rollback and the range of motion was also better, especially in the PS and BCS with postcam mechanism. In the present study, PS and BCS show implant-specific kinematics of rollback induced by postcam, whereas CS, CR, and BCR might have different kinematics due to ligament balancing. The CS, CR, and BCR have different kinematics depending on ligament balancing as well as implant-specific kinematics [21–23]. In the future, it is desirable to improve surgical techniques to achieve deep knee flexion and medial and lateral rollback in those implants.

The limitations of this study are as follows: (1) this study was a retrospective study, and there was bias in data and implant selection. Although there was a data selection bias because there were 40 knees in the fluoroscopic analysis for which PROs could not be obtained in the study period, the reliability of the data was considered to be high because of the large data set compared to past fluoroscopic analyses. Although implant selection was based on the degree of deformity, various factors, such as preoperative range of motion, alignment, and pain, were included in the results. However, when various implants and preoperative conditions were added as factors, the results showed that the postoperative flexion angle was an important factor in improving PROs. (2) Both the postoperative 2011 KSS score and kinematics are expected to be influenced by the preoperative condition; however, these data were not available for this study. Additional research is needed to evaluate the amount of change from preoperative data, which may provide new insights. (3) The present study is based on kinematic analysis only, without kinetic data. The importance of active knee flexion may be further evaluated by including kinetics in the analysis, which is a subject for future study.

## Conclusions

Maximum flexion and lateral rollback are important for better patient satisfaction after TKA. To obtain the maximum flexion angle, it was necessary to perform the normal kinematic pattern with a large amount of lateral rollback. It will be necessary to develop surgical techniques and implants to induce such kinematics in the future.

## Data Availability

Not applicable.
